# Implementation of a behavioral medicine approach in physiotherapy: a process evaluation of facilitation methods

**DOI:** 10.1186/s13012-019-0942-y

**Published:** 2019-11-04

**Authors:** Johanna Fritz, Lars Wallin, Anne Söderlund, Lena Almqvist, Maria Sandborgh

**Affiliations:** 1 School of Health, Care and Social Welfare, Mälardalen University, Box 883, SE-721 23 Västerås, Sweden; 2 School of Education, Health and Social Studies, Dalarna University, Falun, Sweden; 3 Department of Neurobiology, Care Sciences and Society, Division of Nursing, Karolinska Institutet, Stockholm, Sweden; 4 Department of Health and Care Sciences, The Sahlgrenska Academy, University of Gothenburg, Gothenburg, Sweden

**Keywords:** Physiotherapy, Social learning theory, Self-regulation, Implementation science, Knowledge translation, Primary health care, Clinical competence

## Abstract

**Background:**

In a quasi-experimental study, facilitation was used to support implementation of the behavioral medicine approach in physiotherapy. The facilitation consisted of an individually tailored multifaceted intervention including outreach visits, peer coaching, educational materials, individual goal-setting, video feedback, self-monitoring in a diary, manager support, and information leaflets to patients. A behavioral medicine approach implies a focus on health related behavior change. Clinical behavioral change was initiated but not maintained among the participating physiotherapists. To explain these findings, a deeper understanding of the implementation process is necessary. The aim was therefore to explore the impact mechanisms in the implementation of a behavioral medicine approach in physiotherapy by examining dose, reach, and participant experiences.

**Methods:**

An explorative mixed-methods design was used as a part of a quasi-experimental trial. Twenty four physiotherapists working in primary health care were included in the quasi-experimental trial, and all physiotherapists in the experimental group (*n* = 15) were included in the current study. A facilitation intervention based mainly on social cognitive theory was tested during a 6-month period. Data were collected during and after the implementation period by self-reports of time allocation regarding participation in different implementation methods, documentation of individual goals, ranking of the most important implementation methods, and semi-structured interviews. Descriptive statistical methods and inductive content analysis were used.

**Results:**

The physiotherapists participated most frequently in the following implementation methods: outreach visits, peer coaching, educational materials, and individual goal-setting. They also considered these methods to be the most important for implementation, contributing to support for learning, practice, memory, emotions, self-management, and time management. However, time management support from the manager was lacking.

**Conclusions:**

The findings indicate that different mechanisms govern the initiation and maintenance of clinical behavior change. The impact mechanisms for initiation of clinical behavior change refers to the use of externally initiated multiple methods, such as feedback on practice, time management, and extrinsic motivation. The lack of self-regulation capability, intrinsic motivation, and continued support after the implementation intervention period were interpreted as possible mechanisms for the failure of maintaining the behavioral change over time.

Contributions to the literature

- The findings indicate that different mechanisms govern the initiation and maintenance of clinical behavior change, which can inform the use of effective implementation methods.
- A combination of the Medical Research Council guidance and social cognitive theory could augment the understanding of implementation processes.
- The design of a process evaluation can aid in elucidating potentially important factors for initiating and maintaining clinical behavior change.

## Background

In a quasi-experimental trial, we used facilitation as the main method to support the implementation of a behavioral medicine (BM) approach in primary healthcare physiotherapy. A large effect size was found (*r* = .72) regarding changes in the physiotherapists’ clinical behavior immediately after the implementation period, but the changes were not maintained at follow-ups [1]. In contrast, self-efficacy in applying the BM approach increased and was maintained at follow-up. To increase understanding of the successful and unsuccessful parts of the intervention, the implementation process warranted further exploration.

Process evaluation has been suggested as an essential part of designing implementation interventions [2, 3]. In the current study, the implementation intervention comprised the methods used to support the implementation of a BM approach. There is growing interest in the use of frameworks and models to make implementation efforts easier to plan and replicate and more likely to succeed by offering a structure and highlighting causal assumptions [4]. The Medical Research Council has provided guidance on how to perform process evaluations of complex interventions [5]. Process evaluations of the implementation of BM interventions in physiotherapy have focused on the fidelity [6, 7] and feasibility [8] of delivery. Process evaluations focusing on impact mechanisms (i.e., how the delivered intervention produces change [5]) are sparse [9–11] and are non-existent in physiotherapy. Thus, there is a need for process evaluations in the physiotherapy context.

A BM approach in physiotherapy is recommended in the treatment of patients with persistent musculoskeletal pain to increase their ability to participate in daily life activities [12–14]. In this study, a BM approach implies a focus on health-related behavior changes in the assessment, analysis, and management of important biopsychosocial factors for behavior change. Behavior change techniques, such as the patient’s goal-setting, self-monitoring of behavior, and feedback on the patient’s behaviors, are important tools [15, 16]. However, the implementation of a BM approach in a real-world setting is complex because of the multiplicity of clinical behaviors that must be adopted [1, 17]. The implementation often results in some changes in knowledge and attitudes, but change in physiotherapists’ traditional biomedical approach is less common [15, 17–22]. Forming new habits also requires considerable time, from 18 to 254 days (median 66 days), for the repetition of behaviors [23]. Further research is needed on how to support the implementation of a BM approach.

Facilitation is a promising strategy to support the implementation of evidence-based guidelines in primary health care [24]. Facilitation involves both the role of a person who facilitates and the process of practices to support the development of new knowledge and skills [25, 26]. The current study applied basic assumptions for behavioral change in social cognitive theory in the facilitation intervention. Social cognitive theory emphasizes that a behavior is reciprocally influenced by personal and contextual factors [27]. Self-regulation is the capability to control and manage these factors [28]. Forethought capability (i.e., the capability for intentional actions), self-efficacy beliefs, self-monitoring, social support, and observational learning are important sources of self-regulation capability for behavior change [27, 29, 30]. By addressing theory-based assumptions in the facilitation intervention, positive outcomes were expected in terms of the physiotherapists’ clinical behavior changes [31].

To explain the findings in the quasi-experimental trial, a deeper understanding of the implementation process was necessary. Thus, the aim of this study was to explore the impact mechanisms in the implementation of a behavioral medicine approach in physiotherapy by examining dose, reach, and participant experiences.

## Methods

### Design

A mixed-methods design [32] was used to explore the implementation process in the experimental group as part of a quasi-experimental trial. The Medical Research Council guidance for process evaluations [5] was used. The guidance emphasizes that the impact mechanisms of the implementation should preferably be linked to the causal assumptions of the intervention, the contextual factors, and the outcomes. With regard to this study, the impact mechanisms for clinical behavior change are linked to assumptions in social cognitive theory and physiotherapists’ integration of the implementation intervention in a primary health care context. The Standards for Reporting Implementation studies (StaRI) [3] was used to report this study (see Additional file 1).

### Participants and setting

All primary health care physiotherapists in three county councils were asked to participate in the quasi-experimental trial [1]. Fifteen physiotherapists were included in the experimental group and 9 in the control group. All physiotherapists in the experimental group (5 male and 10 female, median age 37 years) working at 7 clinics were included in this process evaluation study. The number of participating physiotherapists at each clinic varied between 1 and 4, corresponding to 100% of the physiotherapists at three of the clinics and 25–80% of the physiotherapists at the other 4 clinics. One physiotherapist was the only participant at that clinic and was therefore encouraged to collaborate with another clinic during the implementation period. Participation was voluntary, and all participants gave written informed consent after receiving oral and written information. The clinics received financial reimbursement corresponding to the physiotherapists’ wage costs for the time spent on the project. The physiotherapists were representative of Swedish physiotherapy primary health care in that direct access to physiotherapy was possible, they often represented the first point of contact for patients, and they had high autonomy in relation to other health care professionals. During the implementation, the clinics were fully staffed with regard to physiotherapists.

The physiotherapists’ expectations of their potential for clinical behavior change when participating in the study were rated at a median of 6 (0 = not at all, 10 = to a very high extent). Their expectations of the ability of the BM approach to increase patients’ ability to participate in daily life activities were rated at a median of 8.

### The implementation intervention

Facilitation was chosen as the main implementation intervention. It consisted of an individually tailored multifaceted intervention [1] (see Table 1). The first author acted as the facilitator. She had extensive experience in teaching as well as knowledge and skills of the BM approach and the use of behavior change techniques. Eight facilitation methods were offered by the facilitator to support the physiotherapists (see Table 1). The selection of the facilitation methods was influenced by pre-trial performance [33], previously identified determinants for using the BM approach [34], and assumptions regarding behavior change originating from social cognitive theory [27, 30]. The Behaviour Change Technique Taxonomy [35] was used to describe the active behavior change components in the facilitation methods. For a detailed description of the relationship between the determinants, assumptions based on social cognitive theory, facilitation methods, and behavior change techniques, see Additional file 2. Seven days spread over a 6-month period were allocated for the physiotherapists to participate in facilitative activities during the implementation. Six months was estimated as a reasonable duration for enabling repetitions of behaviors.

**Table 1:** Description of the facilitation methods offered by the facilitator to support the physiotherapists

Facilitation methods	Description
Outreach visits	Ten 2-h outreach visits with the facilitator and participating physiotherapists.
Peer coaching	Both formal (outreach visits) and informal discussions with other participating physiotherapists at the clinic.
Educational materials	Nine web lectures (12–25 min) describing the core components of the behavioral medicine approach and evidence supporting the behavioral medicine approach; 10 video-recorded role-plays (5–13 min); assistive written materials such as a printable diary for patients’ self-monitoring; and a book describing the model for the systematic application of a behavioral medicine approach to physiotherapy clinical practice.
Individual goal-setting	Set from one outreach visit to the next coming. Also including behavioral contract.
Video feedback	Video recordings of their own sessions with patients as a basis for feedback and discussions during the outreach visits.
Self-monitoring in a diary	Self-monitoring of the physiotherapists’ behaviors connected to their individual goals.
Manager support	Manager support by two telephone calls from a researcher during the implementation period to prompt managers’ supportive attention to the participating physiotherapists.
Information leaflet to patients	Information leaflet to patients about what was planned to happen during the physiotherapy session.

### Data collection

Data were collected concerning dose (how much of the implementation intervention was delivered), reach (the extent to which the physiotherapists came into contact with the implementation intervention), and the physiotherapists’ experiences. Dose and reach were measured through individual time allocated for the use of the different facilitation methods, reported by the physiotherapists every second week during the implementation period as “none,” “half an hour,” “an hour,” or “one and a half hours.” The facilitator took notes regarding the physiotherapists’ attendance and the use of video feedback during the outreach visits. Individual goals were documented in free text every second week, and goal achievement was self-reported as “yes” or “no.”

The physiotherapists’ experiences of the implementation process were explored in semi-structured individual interviews conducted four times during and once immediately after the implementation period for a total of five time points. The interview guide was structured using the Medical Research Council guidance for process evaluations [5] covering the physiotherapists’ experiences of the implementation intervention and contextual factors (see Additional file 3). During the last interview, the physiotherapists were asked to identify the five most highly valued facilitation methods and to rank them from one to five, with five being the most valuable. All interviews during the implementation period were conducted by the first author (three by telephone and one in the clinic) and lasted 5–15 min. The telephone interviews after the implementation period were conducted by a researcher not actively engaged in the implementation intervention; these interviews lasted 30–60 min. All interviews were digitally audio-recorded.

### Data analyses

Descriptive statistics were used to analyze how time was allocated for different facilitation methods, the physiotherapists’ attendance in outreach visits, the presence of video feedback, the distribution of individual goals, and the summarized rank scores for the facilitation methods. The IBM Statistical Package for the Social Sciences (SPSS) version 24 was used for statistical analysis.

An inductive content analysis [36] was used to analyze the interviews. The audio-recorded interviews conducted during the implementation period were played several times, and notes were taken and coded. The interviews conducted after the implementation period were transcribed verbatim and read several times for familiarization. Sensitive meaning units were identified, coded, grouped into categories according to similarities, and mapped to the clusters of the Behaviour Change Techniques Taxonomy [35]. The taxonomy includes 93 behavior change techniques grouped into 16 clusters. Categories that did not fit into the taxonomy formed new categories based on the principles of inductive content analysis [36]. The analysis was performed by the first author (JF) and was regularly discussed and validated by the other authors until consensus was achieved.

## Results

### Dose and reach

**Fig. 1:**
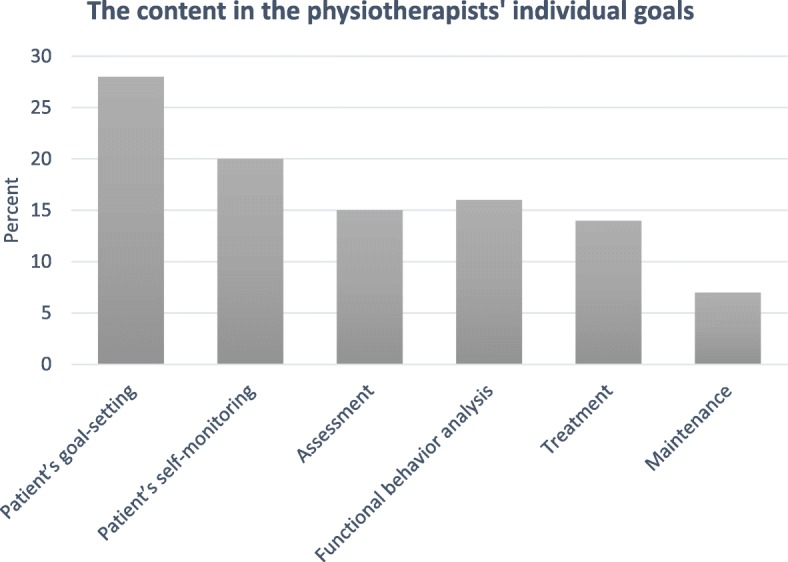
The content of the physiotherapists’ individual goals (*N* = 196), related to the components in the behavioral medicine approach

The physiotherapists self-reported that they used most of the facilitation methods. Outreach visits and individual goal-setting were used to the highest extent, followed by peer coaching and educational material (see Table 2). The documented individual goals represented all components of the BM approach (see Fig. 1). The physiotherapists reported that they achieved 59% of these goals. The educational material consisted of several methods used to varying extents. The interviews revealed that half of the physiotherapist group (*n* = 7) read the entire book, and the other half (*n* = 7) used it as a reference book. Many of the physiotherapists watched all web lectures (*n* = 11) and video-recorded role plays (*n* = 9), and 9 of them watched them several times. The use of the assistive written material ranged from “tried one document” to “used all documents several times.” Video feedback and self-monitoring in the form of a diary were used to the least extent.

**Table 2:** Median values of self-reported dose and reach for the facilitation methods

	Dose median (min–max)	Reach (*n* = 15)
Facilitation methods used during outreach visits	Number of visits	
Participating in outreach visits	9 (3–10)	15
Setting individual goals	9 (3–10)	15
Using video feedback	1 (0–3)	11
Facilitation methods used between outreach visits	Time spent (hours)	
Peer coaching	3 (0–9.5)	12
Educational material	7 (2–14)	15
Self-monitoring through a diary	1 (0–4.5)	7

In addition to these methods, all managers received two telephone calls during the implementation period to remind them about the implementation and to pay supportive attention to the participating physiotherapists. One physiotherapist used the patient information leaflet.

### The physiotherapists’ experiences of the facilitation intervention

The physiotherapists ranked outreach visits as the most valuable facilitation method, followed by peer coaching and educational material (see Table 3).

**Table 3:** The physiotherapists’ summarized ranking points on perceived value of the facilitation methods, range 0–75, 0 = least valuable, 75 = most valuable. (*N* = 15)

Facilitation method	Summarized ranking points
Outreach visits	62
Peer coaching	30
Educational material	
-Web-lectures	24
-Assistive written material	19
-Book	14
-Video-recorded role-plays	14
Video feedback	10
Individual goal-setting	5
Self-monitoring through a diary	0
Manager support	0
Patient information leaflet	0

The interviews revealed that the physiotherapists’ experiences of the facilitation methods reflected seven clusters of behavior change techniques [35] (see Table 4). The following results are presented using the clusters as headings.

**Table 4:** The categories of the physiotherapists’ experiences of the facilitation methods, organized within clusters of behavior change techniques [35]

Clusters of behavior change techniques	Categories of the physiotherapists experiences (linked to facilitation methods)
Goals and planning	Tailored support (outreach visits) Self-management support (individual goals)
Feedback and monitoring	Feedback on clinical practice (outreach visits, peer coaching) Reflection on clinical practice (peer coaching, video feedback, diary) Resistance to use self-monitoring to support clinical practice (video feedback, diary)
Social support	Problem solving when practicing the behavioral medicine approach (outreach visits, peer coaching) Emotional support (outreach visits, peer coaching) Time management support (and lack of) (outreach visits, manager support)
Shaping knowledge	Multiple learning support (educational material, outreach visits)
Comparison of behavior	Role models practicing the behavioral medicine approach (educational material, peer coaching)
Associations	Memory support (outreach visits, individual goals, diary)
Repetition and substitution	Practicing behavior change techniques on themselves (individual goals)

#### Goals and planning

##### Tailored support

The physiotherapists emphasized that it was important that the facilitator was able to tailor the facilitation to the physiotherapist’s knowledge and skills and contextual factors related to the workplace.

“She has been receptive and notices where we are now and what we need”. (Physiotherapist 24)

##### Self-management support

The individual goals helped the physiotherapists’ self-management of action planning by focusing and concretizing the physiotherapists’ practice of BM-related skills between the outreach visits. Two physiotherapists found that the importance of reaching and reviewing the individual goals became secondary when there was a lack of time and suggested stricter follow-ups on goal fulfillment.

“It is a great way to limit and focus that this is what I will do until the next time. You get a clear task”. (Physiotherapist 24)

#### Feedback and monitoring

##### Feedback on clinical practice

Outreach visits and peer coaching provided the opportunity to receive feedback from both an experienced person such as the facilitator and someone in the same position as oneself. Discussing the application of the BM approach also highlighted the progression.

“When you talk about how it was, then you see that it has moved forward. It has been beneficial. You can see that things happen. You think that nothing happens, but it does”. (Physiotherapist 18)

##### Reflection on clinical practice

Peer coaching stimulated reflection when the physiotherapists explained to others what they had practiced and why. The physiotherapists realized that watching themselves in a video-recorded session stimulated reflections that increased their self-awareness of clinical behavior. Those who tried self-monitoring through a diary also found that it contributed to reflections on clinical behavior.

”[The diary] has contributed to learning by making you pause and reflect and write it down”. (Physiotherapist 10)

##### Resistance to using self-monitoring to support clinical practice

There was resistance among the physiotherapists towards using the tools for self-monitoring of their own practice in the BM approach. They felt uncomfortable about showing video recordings of themselves at the outreach visits, the preparation was considered time-consuming, and some patients did not want to be video-recorded. The diary contained several parts that the physiotherapists found confusing, which made the diary too complicated to use in relation to perceived gains. It was also easy to forget to write in the diary, especially when there was a shortage of time.

“It was difficult with all the terms that you are not really used to and to know what everything meant. Have I done that or have I not done it? It was a bit unclear”. (Physiotherapist 6)

#### Social support

##### Problem-solving when practicing the BM approach

Outreach visits and peer coaching contributed to problem-solving when using the BM approach. The exchange of experiences, both with the facilitator and with peers, stimulated problem-solving through discussions of difficulties and by providing ideas for handling these problems. These discussions facilitated new ways of thinking regarding the BM approach and stimulated reflection and understanding.

“There is someone else from outside who has a lot of experience in the work method and can do it well. She asks some questions: ‘Could you have done otherwise? What if you did this? Can you think like this?’ She does it in a different way than we have done”. (Physiotherapist 18)

##### Emotional support

Outreach visits and peer coaching also contributed to the confirmation of emotions from peer physiotherapists when implementing the BM approach.

“It was good to hear that there were others who had difficulties with how to document … ”. (Physiotherapist 20)

##### Time management support (and lack of)

The physiotherapists perceived that the outreach visits provided a structure for time management during the implementation period. Frequent visits (every second week) facilitated behavioral change. Having these visits in the clinic saved time and the fixed time points ensured that this occurred. Three physiotherapists found the outreach visits to be scheduled too tightly and felt that they did not leave time for practicing the BM approach between the visits. They were aware that it was their responsibility to allocate time, but they perceived time to be scarce. The physiotherapists needed a structure for time allocation and scheduled time for using the implementation methods to support changes in practice.

All physiotherapists except one did not recognize active time management support from the manager. One clinic recruited extra staff during the implementation period to reduce the physiotherapists’ workload. Although the physiotherapists knew that they had permission from the manager to allocate time for the implementation, it was challenging to prioritize this before patient care. They did not feel that they received any practical support in prioritizing their daily work to allow them to also have time for the BM education.

“[The manager] has not continuously asked, ‘How is it going? How do you manage to allocate time? How does it fit with the daily work? Is there anything I can do to support you? I think you can try to prioritize this and this so you can focus on the education during this time’. I would have liked more support”. (Physiotherapist 10)

#### Shaping knowledge

##### Multiple learning support

The interviews revealed that the physiotherapists preferred multiple learning methods. Web lectures provided an overview of how to apply the BM approach, but they did not provide the possibility to ask questions and interact with the lecturer, which was highly valued during the outreach visits. The physiotherapists said that the book usefully illustrated the integration between theory and the application of the BM approach in clinical cases. The theoretical part was sometimes difficult to grasp because the physiotherapists felt that it contained uncommon theoretical concepts. During the outreach visits, they were able to discuss these concepts and obtain explanations that developed their BM knowledge. Three physiotherapists did not feel the need to read the book because they had recently read it in their undergraduate education or had dyslexia and difficulties with written information. The assistive written material contributed to clarity and standardization when applying the BM approach. Web-based educational materials were perceived to enable flexibility of time and repetition.

“For me, it's easier with a book to scroll in. I would rather read the book than look at web lectures”. (Physiotherapist 26)

“I did not learn so much from reading the book … .I learned more from web lectures. But that's the way I learn”. (Physiotherapist 6)

#### Comparison of behavior

##### Role models practicing the BM approach

The peer physiotherapists and the video-recorded role-plays functioned as role models when implementing the BM approach. The interviews showed that discussing clinical experiences using the BM approach with peer physiotherapists provided inspiration and clinical solutions. The video-recorded role-plays gave the physiotherapists examples of what to say and do when practicing the BM approach.

“It has been a lot about what everyone has done and what has been tested. One can hear, as an inspiration for others, what they had done, and then you can copy those ideas. At the same time, if you encounter problems, you can get help. What did my colleague do in that situation? I have learned a lot from it”. (Physiotherapist 16)

Three physiotherapists were annoyed that the video-recorded role-plays were simulated, which made it more difficult for them to see the connection to reality. Three other physiotherapists reported that the video-recorded role-plays were uninspiring and too long.

#### Associations

##### Memory support

According to the physiotherapists, the outreach visits and the individual goals acted as reminders to practice the required skills for a BM approach. The physiotherapists said that they did their homework so that they had something to discuss with the facilitator when she arrived. Additionally, self-monitoring through a diary helped the physiotherapists remember the cases they wanted to discuss during the outreach visits to obtain feedback.

“If I have written down that I will do these things, then it will be done. At least for me. Then you have it there and then you try a little harder, and it works. For me, it is a spur to do so”. (Physiotherapist 18)

#### Repetition and substitution

##### Practicing behavior change techniques on themselves

Goal-setting is both a facilitation method to support the implementation of the BM approach and an important behavior change technique within the BM approach. By setting their own individual goals during the implementation period, the physiotherapists practiced goal-setting on themselves. Their own experiences of practice contributed to developing their goal-setting skills.

“It was useful to see that this is also the case for patients if they set goals that are too high. They will not reach them, and they will be disappointed. It is much better to set lower goals to be able to reach them from one session to the next”. (Physiotherapist 8)

### Context

Some experiences were not related to the facilitation methods or the clusters of behavior change techniques but rather concerned important contextual factors for the implementation.

#### High workload

The physiotherapists perceived the workload to be high. All participating clinics had a waiting list for physiotherapy with a median waiting time of 3–4 weeks (range 1–6 weeks). Because of this, the time for preparation and reflection was reduced. During the implementation period, 12 out of 15 physiotherapists participated in other courses with a median duration of 4 days (range 2–15 days). Five of these physiotherapists found that these courses concurred with the implementation regarding both time and engagement.

#### Lack of keywords for psychosocial and behavioral perspectives

An important task for physiotherapists is to document their treatments and conclusions in the patient record. Half of the physiotherapists stated that the patient record system could have prompted the BM approach if the key words had included a psychosocial and behavioral perspective to a greater extent. The keywords could then have served as reminders to include these perspectives.

## Discussion

Because of high workload, the physiotherapists reported difficulties in prioritizing time for the implementation. Although the physiotherapists knew that they were allowed to allocate time for the implementation, they needed support for this. The outreach visits scheduled by the facilitator contributed to a structure that supported the allocation of time for implementation. A lack of time was mentioned in this and other studies as a barrier to implementation [34, 37, 38] that hindered the repetition required to establish habits [23]. The physiotherapists perceived that support from the manager in prioritizing their daily work was a prerequisite for implementation. All managers were encouraged to actively support the physiotherapists during the implementation, but only one physiotherapist perceived that this happened. Tistad et al. [10] found that managers needed support to develop leadership behaviors in operationalizing the implementation plans. Aarons et al. [39] noted that paying attention to implementation and allocating resources are important for managers’ facilitation of strategic climates for implementation. The challenge for many managers is to find the time to coach. Managers with a small number of employees (which was not the case in the clinics included in our study) appear to have better opportunities for coaching [40]. When implementing new methods, time management is important. An external facilitator can contribute to this support during the implementation intervention period, but an engaged manager contributes to more sustainable support.

Role models provided by the video-recorded role-plays contributed to support for practice through observational learning. Most of the video-recorded role-plays concerned the same BM components as the areas in which a change occurred in the physiotherapists’ clinical behavior [1]. If video recordings of the other components of the BM approach had been provided, increases in these clinical behaviors might have been found. Observational learning can be a shortcut when learning new behaviors [27] and can be helpful in a time-pressed work situation.

Social influences such as peer coaching can increase physiotherapists’ capacity to initiate and maintain behavioral change [27, 41]. Peer coaching contributed to support for practice through feedback, reflection and problem solving, and emotional support. These results are consistent with previous research suggesting that interventions focusing on action, experience, and peer support are more likely to lead to professional behavior change in health care [11]. Learning new behaviors is linked to feelings of anxiety and frustration that require emotional support [42]. Emotional support can also be important to overcome feelings of embarrassment when asking about psychosocial factors [34]. However, there are barriers to making peer coaching work in reality. The physiotherapists had difficulty arranging peer-coaching situations and needed the facilitator to schedule a time for these situations.

The use of individual goals and behavioral contracts functioned as self-management support to structure the skills training as part of the forethought capability [29]. The physiotherapists did not rate their own individual goal-setting as an important implementation method, which was contradicted by their experiences of goal-setting as a cue for skills training. The majority of the goals that were set during the implementation period corresponded to the same BM components for which a change in clinical behavior was found. It seems that these components were practiced to a larger extent than the components for which no change in clinical behavior was found. Locke and Latham [43] claim that goal-setting affects motivation and persistence in achieving the goal. Given that the goal achievement in this study was only 59%, this phenomenon was not observed. The physiotherapists’ motivation may have been extrinsically driven by a willingness to please the facilitator. Behavior change is more likely to be maintained if the person perceives intrinsic motivation, such as satisfaction in performing the activity itself [41, 44]. Most activities performed by physiotherapists in the clinic are not intrinsically motivated but rather are performed to achieve patient outcomes or to comply with guidelines and regulations. Nevertheless, self-rewards and self-control can contribute to a sense of competence and autonomy that is important for enhancing intrinsic motivation [44]. Thus, the stimulation of self-reinforcement through satisfaction with goal achievement can be a successful method to increase intrinsic motivation for the maintenance of behavior change.

Different combinations of facilitation methods were preferred by different physiotherapists, revealing variation in their preferred ways of learning to acquire knowledge and skills. In higher education, multiple learning methods that integrate web-based and face-to-face learning activities have positive effects on students’ learning [45]. An intervention including both practical tools and the ability to ask questions and receive feedback from a facilitator has a positive impact on learning outcomes [18]. Thus, facilitation should be tailored to the physiotherapist’s personal preferences. In our study, the facilitation was tailored in relation to adaptations of the action plan, problem-solving, and reflections based on the physiotherapists’ needs. It is possible that an even more tailored intervention would have had a greater impact on the outcomes. The challenge is to balance adaptations of the implementation intervention for both the individual physiotherapist and the group of physiotherapists working at the same clinic.

Self-monitoring by video or diary was not widely used by the physiotherapists, thus excluding self-monitoring as support for the practice in our study. These self-monitoring methods aim to stimulate attention to one’s own performance as a self-diagnostic function prior to goal-setting and to stimulate the self-motivating function through reinforcement. Although self-monitoring is important for supporting behavior change [29, 46], it was of little prominence as the physiotherapists did not use it. Thus, there is a need to identify other feasible methods for self-monitoring. It is possible that to be able to manage the barriers to using video recordings, stronger emotional support is required than was offered in this study.

The physiotherapists in our study asked for keywords in the patient records for psychosocial and behavioral factors that could function as reminders to use the BM approach. Computer reminders in electronic patient records have been used as memory support to prompt new behaviors [47]. Small to modest improvements were found, but these improvements were larger when a response from the user was required to proceed. According to these results, computer reminders alone would probably not change the physiotherapists’ behavior. However, as part of a multifaceted implementation strategy, a computer reminder requiring an answer from the physiotherapist may contribute to forming habits and thus to the maintenance of the behavior change [41].

The decision to initiate a behavior change depends on expectations regarding future favorable outcomes [48]. The physiotherapists had high expectations regarding patient outcomes when using the BM approach, but the perceived importance of using each BM component was moderate [1]. The core components “patients’ goal-setting,” “promoting patients’ self-monitoring,” and “functional behavior analysis” were perceived as less important. The physiotherapists’ attitudes towards the BM approach likely affected their intention to use it.

According to social cognitive theory [27, 30], self-efficacy is a crucial determinant of the initiation and maintenance of behavior change. We previously reported increased self-efficacy for using the BM approach as an intermediate effect of the facilitation intervention [1]. Although self-efficacy increased and was maintained, the behavioral changes were not maintained [1]. According to Rusk et al. [49], an intervention needs to address multiple domains, helping the system to “tip over” and change. In addition, multiple pathways for change contribute to maintenance through synergistic effects. Both the outreach visits and the individual goal-setting prompted skills training of the BM approach, which likely contributed to synergistic effects due to mastery experience and increased self-efficacy. However, when the external support for implementation ceased, the synergistic effects ended as well. The physiotherapists’ self-efficacy alone seemed insufficient to provide the tip-over effect for clinical behavior change to be maintained. It is therefore important to ensure that synergistic effects can continue after the implementation intervention.

The facilitation intervention addressed several behavioral change techniques [35] described in Additional file 2. The results showed that the physiotherapists perceived most of these techniques as present in the intervention. However, the balance between the facilitation methods is worth considering to maintain clinical behavior change over time. To initiate clinical behavior change in the current study, a variation of externally initiated facilitation methods seemed important. According to Clark and Zimmerman [28], external support should gradually decrease as the self-regulation capability increases. The balance between external support for clinical behavior change and support to increase self-regulation capability is an important factor to consider in future studies.

Well-known theoretical approaches to behavior change [27, 46] do not formally distinguish between how to initiate and how to maintain behavior change. However, our results indicate that different processes guide the initiation and maintenance of behavior change. Theoretical explanations for the maintenance of behavior change focus on motives, self-regulation, habits, resources, and environmental and social influences [41], which correspond to the physiotherapists’ experiences in our study. Therefore, future process evaluation studies should include important factors for maintenance.

### Strengths and limitations

A particular strength of our study lies in the theoretical base. The Medical Research Council guidance for process evaluation [5] provided a structure for exploring the implementation process, and social cognitive theory [27, 29, 30] guided the understanding of the process evaluation findings. This study also concretized and discussed how the components of the social cognitive theory were addressed, which strengthens the transparency of the study. To the best of our knowledge, this is the first study to use social cognitive theory to seek explanations for the underlying processes that make implementation interventions effective. The Behaviour Change Taxonomy [35] was used to clarify and provide further transparency regarding which behavior change techniques were used in the implementation intervention.

Qualitative data about the physiotherapists’ experiences of the facilitation methods complemented the quantitative data about the dose and reach of each method. This information enhanced the understanding of the successful and unsuccessful parts of the implementation intervention. However, the mapping to the Behavior Change Taxonomy [35] was somewhat problematic. There is some overlap between behavior change techniques. Some techniques are described as processes (e.g., feedback on behavior, prompt/cues) and others as strategies to obtain these processes (e.g., social support, goal-setting). To address these overlaps, the physiotherapists’ experiences were categorized in relation to the behavioral change technique that they primarily addressed. The qualitative analysis was continuously discussed and confirmed among the researchers in the study to strengthen the trustworthiness. Quotes from participating physiotherapists are presented to add transparency and trustworthiness to the findings [50].

The characteristics of the sample in this study are likely similar to physiotherapists in primary health care in Sweden, although descriptive studies of primary health care physiotherapy in Sweden or other countries are sparse [51]. The sample in the current study had a wide span in age and work experience and represented both cities and smaller towns. The self-selecting nature of the sample could imply that these physiotherapists were more motivated towards behavior change. However, the physiotherapists’ moderate expectations of changing their clinical behavior by participating in the study suggests the opposite. The contextual factors, such as a high workload and lack of time, are probably valid for most physiotherapists in primary health care. The characteristics and contextual factors taken together thus support the transferability of the findings to physiotherapy in primary health care.

Our results indicate that different processes guide the initiation and maintenance of behavior change [52]. In this study, data were only collected during and immediately after the implementation intervention period. We recommend that future studies extend the duration of the process evaluation to focus on mechanisms for the maintenance of changed behavior.

## Conclusions

This study sheds light on the underlying processes when facilitation is used to support the implementation of a BM approach in physiotherapy. The findings indicate that different mechanisms govern the initiation and maintenance of clinical behavior change. The impact mechanism for the initiation of clinical behavior change refers to the use of externally initiated multiple methods, such as feedback on practice, time management, and extrinsic motivation. The lack of self-regulation capability, intrinsic motivation, and continued support after the implementation intervention period were possible reasons for the failure to maintain the behavioral change over time. The results revealed that outreach visits, peer coaching, educational material, and individual goal-setting were useful facilitation methods to initiate clinical behavior change. To achieve a successful implementation over time, we suggest that these facilitation methods be combined with support for self-regulation capability and intrinsic motivation. The design of process evaluations should include potentially important factors for both initiating and maintaining clinical behavior change.

## Supplementary information

**Additional file 1:** Standards for Reporting Implementation Studies: the StaRI checklist for completion.

**Additional file 2:** Selection of behavior change techniques applied in the facilitation methods to target facilitators and barriers for using the behavioral medicine approach, and concepts related to the social cognitive theory.

**Additional file 3:** Interview guide.

## Data Availability

The data and materials used are available from the corresponding author upon reasonable request, which maintains all participants’ anonymity.

## Abbreviations

BM: Behavioral medicine
SPSS: Statistical package for the social sciences
StaRI: The standards for reporting implementation studies
