# Supplementary material for: Implementation of a behavioral medicine approach in physiotherapy: a process evaluation of facilitation methods
Source: Implement Sci. 2019 Nov 4;14:94. doi: 10.1186/s13012-019-0942-y (PMC6827232; doi:10.1186/s13012-019-0942-y)
Supplement: Supplementary file 2 — Additional file 2: Selection of behavior change techniques applied in the facilitation methods to target facilitators and barriers for using the behavioral medicine approach, and concepts related to the social cognitive theory. [file 13012_2019_942_MOESM2_ESM.docx]

Additional file 2. Selection of behaviour change techniques [35] applied in the facilitation methods to target facilitators and barriers for using the behavioural medicine approach [34], and concepts related to the social cognitive theory [27, 29, 30].

| **Social cognitive theory** | **Facilitators (+) and barriers (-)** | **Facilitation methods** | **Behaviour change techniques** |
| --- | --- | --- | --- |
| Social support  Self-efficacy beliefs | External support (+)  Behavioural medicine knowledge (+)  Ambivalence towards the behavioural medicine approach (+/-)  Embarrassment asking about psychosocial factors (-)  Incomplete skills for applying a behavioural medicine approach (-)  Biomedical instead of biopsychosocial focus (-) | Outreach visits | 1.4 Action planning  2.2 Feedback on behaviour  3.2 Social support (practical)  3.3 Social support (emotional)  4.1 Instruction on how to perform a behaviour  7.1 Prompts/cues  8.7 Graded tasks  10.4 Social reward  11.2 Reduce negative emotions  15.1 Verbal persuasion about capability |
| Observational learning  Social support  Self-efficacy beliefs | Support from peers (+)  Embarrassment asking about psychosocial factors (-)  Incomplete skills for applying a behavioural medicine approach (-) | Peer coaching | 2.2 Feedback on behaviour  3.2 Social support (practical)  3.3 Social support (emotional)  6.2 Social comparison  10.4 Social reward |
| Observational learning | Behavioural medicine knowledge (+)  Lack of time (-)  Ambivalence towards the behavioural medicine approach (-)  Incomplete skills for applying a behavioural medicine approach (-)  Biomedical instead of biopsychosocial focus (-) | Educational material | 4.1 Instruction on how to perform a behaviour  4.2 Information about antecedents  6.1 Demonstration of the behaviour  6.2 Social comparison |
| Forethought capability  Self-efficacy beliefs | Incomplete skills for applying a behavioural medicine approach (-) | Individual goalsetting | - 1. Goal setting (behaviour)   2. Problem solving   1.4 Action planning  1.5 Review behaviour goal(s)  1.8 Behavioural contract  7.1 Prompts/cues  8.1 Behavioural practice/ rehearsal |
| Forethought capability  Self-monitoring | Lack of self-awareness of professional practice (-) | Video feedback | 2.2 Feedback on behaviour  2.3 Self-monitoring of behaviour |
| Forethought capability  Self-monitoring | Lack of self-awareness of professional practice (-) | Self-monitoring in a diary | 2.3 Self-monitoring of behaviour  7.1 Prompts/cues |
| Social support | Lack of support from manager (-) | Manager support | 3.2 Social support (practical) |
| Contextual factors | Patients as active and passive agents (+/-)  Patients’ role expectations of the physiotherapist (+/-) | Patient information | 12.2 Restructuring the social environment |
