# Supplementary material for: Implementation of a behavioral medicine approach in physiotherapy: a process evaluation of facilitation methods
Source: Implement Sci. 2019 Nov 4;14:94. doi: 10.1186/s13012-019-0942-y (PMC6827232; doi:10.1186/s13012-019-0942-y)
Supplement: Supplementary file 3 — Additional file 3: Interview guide. [file 13012_2019_942_MOESM3_ESM.docx]

# Interview guide

**Tell me about your experiences of applying the behavioural medicine approach.**

- What made it easier/more difficult?
- Do you have any thoughts on how to deal with the difficulties?

**Tell me about your experiences of the implementation period and each facilitation method.**

- Something which was particularly good?
- Something you wish would have been done differently?
- To what extent have you used the different facilitation methods (e.g. read the whole book or parts of it)?
- In what way have these [facilitation methods] contributed to your learning?
- How will you manage to maintain your new knowledge and skills?

**Contextual factors**

How long is your waiting list? How does the waiting list affect the implementation of the behavioural medicine approach?

What other courses did you attend during the implementation period? Number of training days? How did these courses influence the implementation of the behavioural medicine approach?

How do you perceive the patient record system in relation to the behavioural medicine approach? Hindering/supporting?
